# L-ficolin and H-ficolin in patients with Familial Mediterranean fever

**DOI:** 10.1186/1546-0096-13-S1-P101

**Published:** 2015-09-28

**Authors:** G Mkrtchyan, A Boyajyan

**Affiliations:** 1 Institute of Molecular Biology of Armenian National Academy of Sciences, Yerevan, Armenia

## 

In the present study we performed comparative determination of the blood levels of L-ficolin and H-ficolin, opsonins and the initial components of the complement lectin pathway, in patients with Familial Mediterranean fever (FMF) in attack and attack-free periods and healthy subjects. In addition, in all study subjects serum levels of IL-1b and and total leukocyte count (TLC) were determined. Fifty FMF-affected subjects (female/male: 25/25; mean age ± SD: 31.3 ± 11; in attack/attack free: 23/27) and 81 age- and sex-matched healthy subjects (control group) without family history of FMF or other autoinflammatory diseases were enrolled in this study. Elevated H-ficolin level and TLC were detected in patients during attack period, whereas increased levels of L-ficolin and IL-1b were found in both attack and attack free patients with higher values during attack. Positive correlation between H-ficolin and L-ficolin levels in patients and healthy subjects was detected. Our results suggest excess production of L- and H-ficolins and increased apoptosis rate in FMF and indicate that H-ficolin is operating during development of acute autoinflammatory reactions, whereas L-ficolin is operating in both acute and subclinical autoinflammatory responses associated with this disease.

